# M^2^HepPrEP: study protocol for a multi-site multi-setting randomized controlled trial of integrated HIV prevention and HCV care for PWID

**DOI:** 10.1186/s13063-022-06085-3

**Published:** 2022-04-23

**Authors:** Valérie Martel-Laferrière, Daniel J. Feaster, Lisa R. Metsch, Bruce R. Shackman, Christine Loignon, Bohdan Nosyk, Hansel Tookes, Czarina N. Behrends, Nelson Arruda, Oluleye Adigun, Marie-Eve Goyer, Michael A. Kolber, Jean-Francois Mary, Allan E. Rodriguez, Iveth G. Yanez, Yue Pan, Rania Khemiri, Lauren Gooden, Aïssata Sako, Julie Bruneau

**Affiliations:** 1 Centre hospitalier de l’Université de Montréal, Montreal, Canada; 2 University of Miami Miller School of Medicine, Miami, USA; 3 Columbia University Mailman School of Public Health, New York City, USA; 4 Weill Cornell Medical College: Weill Cornell Medicine, New York City, USA; 5 Université de Sherbrooke, Sherbrooke, Canada; 6 Simon Fraser University, Burnaby, Canada; 7 Direction régionale de la santé publique de Montréal, Montreal, Canada; 8 Golden Glades Treatment Center, North Miami Beach, USA; 9 Faculté de médecine: Université de Montréal, Montreal, Canada; 10 CACTUS Montréal, Montreal, Canada; 11 University of Miami Department of Public Health Sciences, Miami, USA; 12 Centre de Recherche du CHUM: Centre hospitalier de l’Université de Montréal Centre de Recherche, Montreal, Canada

**Keywords:** Pre-exposure prophylaxis (PrEP), Hepatitis C treatment, People who inject drugs, Patient navigator, Adherence counselor

## Abstract

**Background:**

Opioid use is escalating in North America and comes with a multitude of health consequences, including HIV and hepatitis C virus (HCV) outbreaks among persons who inject drugs (PWID). HIV pre-exposure prophylaxis (PrEP) and HCV treatment regimens have transformative potential to address these co-occurring epidemics. Evaluation of innovative multi-modal approaches, integrating harm reduction, opioid agonist therapy (OAT), PrEP, and HCV treatment is required. The aim of this study is to assess the effectiveness of an on-site integrated care model where delivery of PrEP and HCV treatment for PWID takes places at syringe service programs (SSP) and OAT programs compared with referring PWID to clinical services in the community through a patient navigation model and to examine how structural factors interact with HIV prevention adherence and HCV treatment outcomes.

**Methods:**

The Miami-Montreal Hepatitis C and Pre-Exposure Prophylaxis trial (M^2^HepPrEP) is an open-label, multi-site, multi-center, randomized, controlled, superiority trial with two parallel treatment arms. A total of 500 persons who injected drugs in the prior 6 months and are eligible for PrEP will be recruited in OAT clinics and SSP in Miami, FL, and Montréal, Québec. Participants will be randomized to either on-site care, with adherence counseling, or referral to off-site clinics assisted by a patient navigator. PrEP will be offered to all participants and HCV treatment to those HCV-infected. Co-primary endpoints will be (1) adherence to pre-exposure prophylaxis medication at 6 months post-randomization and (2) HCV sustained virological response (SVR) 12 weeks post-treatment completion among participants who were randomized within the HCV stratum. Up to 100 participants will be invited to participate in a semi-structured interview regarding perceptions of adherence barriers and facilitators, after their 6-month assessment. A simulation model-based cost-effectiveness analysis will be performed to determine the comparative value of the strategies being evaluated.

**Discussion:**

The results of this study have the potential to demonstrate the effectiveness and cost-effectiveness of offering PrEP and HCV treatment in healthcare venues frequently attended by PWID. Testing the intervention in two urban centers with high disease burden among PWID, but with different healthcare system dynamics, will increase generalizability of findings.

**Trial registration:**

Clinicaltrials.gov NCT03981445. Trial registry name: Integrated HIV Prevention and HCV Care for PWID (M2HepPrEP). Registration date: June 10, 201.

**Supplementary Information:**

The online version contains supplementary material available at 10.1186/s13063-022-06085-3.

## Administrative information

**Table:** 

Title	M^2^HepPrEP: A Multi-Site Multi-Setting Randomized Controlled Trial of Integrated HIV Prevention and HCV Care for PWID
Trial registration	NCT03981445
Protocol version	Protocol version 1.0
Funding	NIH NIDA: study funding (R01DA045713) Gilead Sciences*®*^*:*^: Medication
Author details	Centre de recherche du Centre hospitalier de l’Université de Montréal Medical microbiology and infectious diseases service, Centre hospitalier de l’Université de Montréal Microbiology, infectious diseases and immunology, Université de Montréal Mailman School of Public Health Columbia University Department of Sociomedical Sciences Weill Cornell Medical College Department of Population Health Sciences Département de médecine familiale et de médecine d’urgence, Université de Montréal CIUSSS Centre-Sud-de-l’Île-de-Montréal CACTUS Montréal Jackson Memorial Hospital Department of Public Health Sciences, Biostatistics Division, University of Miami Department of Sociomedical Sciences, Mailman School of Public Health, Columbia University Centre hospitalier de l’Université de Montréal Department of family and emergency medicine, Université de Montréal
Name and contact information for the trial sponsor	Guifang Lao, M.D., PhD. Health Scientist Administrator/Program Officer laog@mail.nih.gov [laog@mail.nih.gov]
Role of sponsor	The National Institute on Drug Abuse, the study sponsor, and Gilead Sciences*®*, provider of study medication, are not directly involved in study design, collection, and management analysis of data or writing of reports. They also will not have any role in submission of publication. NIDA does review period summary reports for progress. Gilead does receive medication-related safety data.

## Introduction

### Background and rationale (6a)

The US Centers for Disease Control and Prevention (CDC) has referred to the North American opioid overdose crisis as a “public health epidemic” [1]. Prescription opioid use, heroin use, fentanyl use, and associated substance use disorders are escalating in North America and are responsible for a multitude of health consequences, including recent HIV and hepatitis C virus (HCV) outbreaks among persons who inject drugs (PWID) [2–10]. Studies suggest that injection drug use is associated with 1 in 10 new HIV infections in the USA, 1 in 9 new HIV infections in Canada [11, 12], and with the majority of new HCV infections in both countries [13]. The proportion of HCV disease burden attributable to drug injection is estimated to be 80% [14]. Of PWID who are living with HIV, approximately 80% also have HCV antibodies [15]. Notably, increased use of injection drugs in the USA has driven up the number of estimated acute HCV cases by 400% between 2010 and 2017 [16].

Significant improvements in prevention and care are required to substantially reduce HIV and HCV infections among PWID [17, 18]. Pre-exposure prophylaxis (PrEP) and HCV treatment regimens have transformative potential to address these co-occurring epidemics [19–21]. Integrated HIV prevention services have been recommended for PWID [22–24], and PrEP efficacy for PWID has been shown in a randomized controlled study [25]. In addition, PWID’s interest in PrEP has been confirmed by an international study [26, 27]. The CDC now recommends that all individuals at substantial risk of HIV, including PWID, should be offered PrEP as part of integrated services [28–30]. However, there is insufficient evidence on the optimal method of providing care [31, 32]. Because PWID have competing social needs and comorbidities, effective and efficient service delivery along the care continuum is essential to avoid missed opportunities and disengagement. There is a strong consensus supporting PrEP implementation for PWID in combination with harm reduction and other healthcare interventions [33, 34]. Hence, it is important to study “test, treat and prevent” models of care among PWID that use multi-modal approaches, such as integrating syringe service programs, opioid agonist treatment, and HCV treatment with PrEP, and to evaluate their potential efficacy.

The overall goal of this project is to compare on-site integrated delivery of HIV PrEP and HCV treatment for PWID (HepPrEP) at syringe service programs (SSP) and opioid agonist therapy (OAT) programs to off-site referral to clinical services in the community with patient navigation and to examine how structural and organizational factors interact with HIV prevention and HCV treatment uptake and adherence in two cities (M^2^).

### Objectives (7)

The primary study objective is to compare the effectiveness of on-site integrated care that includes adherence counseling vs. off-site referral to care with support of a patient navigator in increasing rates of (1) PrEP adherence (0–6 months) and (2) HCV sustained viral response 12 weeks post-treatment completion (SVR12) among PWID recruited in OAT and SSP in Miami, FL, and Montreal, Quebec. The study will examine the relative contributions of living in cities with different healthcare systems (Miami vs. Montreal) and venues (OAT vs. SSP) on primary outcomes.

Secondary objectives are to measure differences between study arms in (1) PrEP initiation, (2) long-term sustained PrEP adherence (6–18 months), (3) HCV infection/reinfection, (4) sexually transmitted infections and reinfection (including HIV), and (5) behavioral disinhibition, i.e., the proportion of participants who increase sexual or injection risk behaviors.

The study will include a qualitative component with the following objectives: (1) to explore individual and structural factors influencing PrEP initiation and adherence; (2) to examine how PWID understand and define PrEP adherence; (3) to describe and understand PWID’s experience regarding on-site and off-site interventions including their perceived effects on PWID’s health; (4) to examine if and understand how PrEP use is related to HIV risk behavior modifications among PWID; (5) to understand facilitators and barriers to implementation and how study staff experience and address these factors during the trial.

Finally, a cost-effectiveness analysis will evaluate (1) healthcare services utilization and cost-effectiveness of the on-site integrated care approach compared to the referral with patient navigator and (2) the healthcare resources required to scale up these intervention approaches in the local environments and site settings.

### Trial design (8)

The M^2^HepPrEP trial is an open-label, multi-site, multi-center, randomized, controlled, superiority trial with two parallel treatment arms. The study is a type-1 hybrid effectiveness-implementation study [35, 36]. PrEP eligible participants (+/− eligible for HCV treatment) will be randomized to on-site integrated care with adherence counseling (on-site arm) or off-site referral to PrEP and HCV treatment (if HCV positive) with patient navigation (off-site arm) in a 1:1 ratio using a permuted-block randomization scheme.

A sample of up to 100 trial participants and venue staff will be invited to participate in the qualitative study component. Cost-effectiveness analysis will be performed using data collected in the trial. A computer simulation model will be used to estimate the individual benefits of HCV treatment and the public health benefits of reduced onward transmission of HIV and HCV due to PrEP and HCV treatment.

**Fig. 1:**
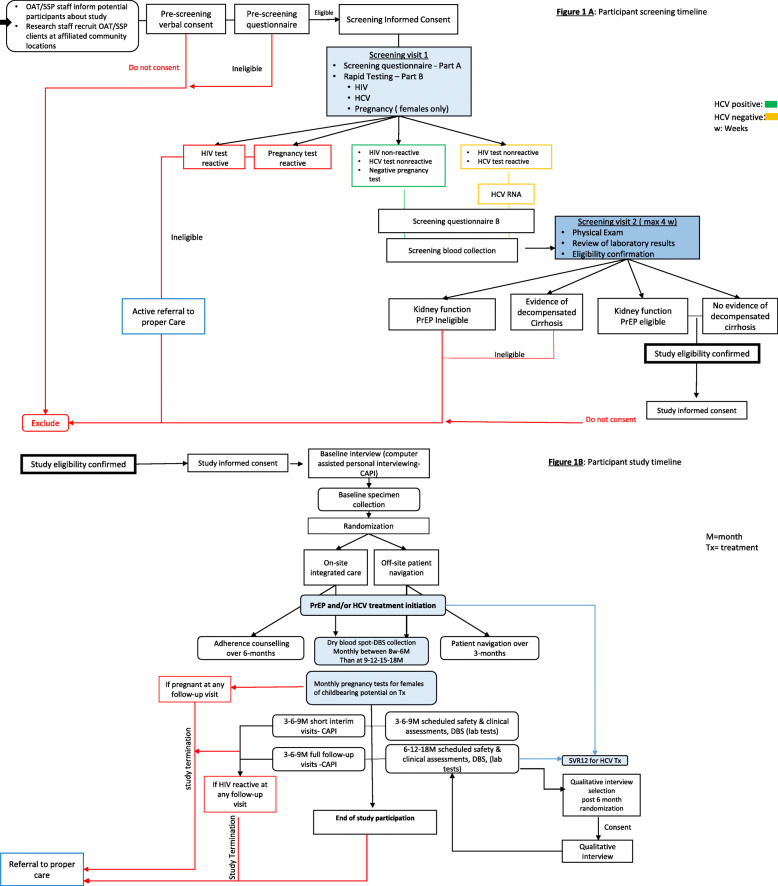
**A** Participant screening timeline. **B** Participant study timeline

The study protocol (version 1.0) follows the Standard Protocol Items: Recommendations for Interventional Trials (SPIRIT) Statement (see Fig. 1 and Additional file 1).

**Table 1:** Sites

Sites	Miami		Montréal	
	Golden Glades Treatment Center	IDEA	Programme Relais du CIUSSS Centre-Sud-de-l’Île-de-Montréal	Cactus Montréal
Type	OAT clinic	SSP	OAT low threshold clinic	SSP
Funding	Private Insurance, Medicaid and Self-Pay	Private donations	Public	Public
Physicians on site	Yes	Weekly	On week day	No
Specialized in addiction	Yes	Yes	Yes	Yes
Number of physicians	2	2	5	0
Number of nurses	7	1	3	0,1
Other resources onsite	Counselling, drug testing, HIV prevention	OAT, wound care, HIV care	Standard and alternative OAT, primary care, provincial medical care card dispensing services, others	STI and hepatitis prevention education, telemedicine OAT clinics, HCV testing, sex worker/transgender person support
Clients per year	250	500–700	200–300	500–700

## Methods: participants, interventions, and outcomes

### Study setting (9)

The study is conducted in Miami, FL, USA, and Montreal, Québec, Canada. In each city, participants are recruited through their affiliation with SSP and/or OAT clinics. OAT is a relatively high-resourced setting with medical monitoring of treatment. SSP, on the other hand, are frequently low resourced, particularly in the southern USA. This environment is rapidly evolving in the context of COVID-19, however, with increased availability of more flexible and low-threshold OAT programs in the US and Canada, including integrating OAT at SSP [37–39] (Table 1).

### Eligibility criteria (10)

Individuals must meet the following criteria to be eligible to participate in the RCT: (1) being 18–64 years of age; (2) report injection drug use in the past 6 months; (3) be HIV negative; (4) provide informed consent; (5) complete a medical release form; (6) report living in the vicinity and being able to return for follow-up over 18 months; (7) be willing to use a medically acceptable form of contraception throughout the study duration (for females of childbearing potential); (8) be able to communicate in English, French, or Spanish (site dependent); and (9) be receiving services at an OAT clinic or SSP.

Individuals are excluded from study if they (1) have any disabling medical conditions as assessed by medical history, physical exam, vital signs, and/or laboratory assessments that, in the opinion of the study physician, preclude safe participation in the study or ability to provide fully informed consent; (2) have any disabling mental conditions as assessed by medical history and clinical assessment that, in the opinion of the study physician, precludes safe participation in the study or ability to provide fully informed consent; (3) have chronic renal failure; (4) have or have history of decompensated cirrhosis; (5) diagnosed with HIV or have symptoms of an acute HIV infection; (6) are pregnant (verified via pregnancy test), are planning to become pregnant during the course of the study, or breastfeeding; (7) have an allergy or contraindication to one of the study medications; (8) have prior HCV treatment failure with direct-acting antiviral (DAA) regimens (except in case of demonstration in medical record of SVR12 followed by reinfection); (9) are currently on PrEP and/or receiving HCV treatment; and (10) are currently in prison or jail, in any inpatient overnight facility as required by court of law or have a pending legal action, which may prevent an individual from completing the study.

### Who will take informed consent? (26a) Additional file 3

Participants are invited to participate in the study either by the service or the study staff while they are receiving services either at the SSP or OAT sites. Both screening and trial informed consent forms are distributed to participants and explained by study interviewers and/or research nurses. Participants are offered time to read, understand, and ask questions regarding the study and the informed consent forms.

### Additional consent provisions for collection and use of participant data and biological specimens (26b)

Whereas no additional studies are planned on this data at this time, any emergent issues affecting the protocol and/or the populations may result in additional studies.

## Interventions

### Explanation for the choice of comparators (6b)

PWID face numerous challenges while trying to navigate the healthcare system and thus PrEP and HCV treatment initiation remains low in this historically disadvantaged population. Patient navigation, either by professionals or peers, has been studied across various health conditions. Navigation has the advantage of being feasible with limited resources and without a complete reorganization of services. Despite its popularity, formal evaluation of this strategy in PrEP and HCV contexts is relatively limited. Studies to date have shown that patient navigation is associated with a modest, but not always significant, improvement in treatment uptake, such as a higher rate of initiation or shorter delays in initiation [40–43]. Navigation helps individuals facing some structural barriers in the healthcare system but is still limited by the need for often marginalized individuals to access care in new settings outside of their regular health or community resources [44, 45]. An alternative strategy to improve access to care is implementation of integrated, on-site services within existing OAT or SSP. However, questions remain about the relative effectiveness and the amount of resources required to provide integrated services on site.

### Intervention description (11a)

#### Pre-screening

Individuals who express interest in participating in the study meet with the study staff and are invited to provide verbal informed consent to complete a brief pre-screening interview which determines their eligibility to move forward to study screening activities. The pre-screening interview is brief and consists of questions about the individual’s sex, race/ethnicity, age, history of injection drug use, and HIV status. Individuals who pre-screen as ineligible do not continue in the study and proceed as they normally would for routine services or treatment.

#### Screening

Individuals who pre-screen as eligible and are interested in participating in the study are asked to provide written consent for the screening process and to complete a consent quiz to demonstrate their understanding of the eligibility requirements. Potential participants provide capillary blood and urine samples to complete a point-of-care HIV test, an HCV test, and a pregnancy test (for persons of childbearing potential). Venipuncture and urine samples are collected for lab tests that will further assess HIV status, HCV status, and test hepatitis B virus (HBV) status, liver function, and kidney function (Table 2). Participants complete 5 questionnaires to assess eligibility (Table 3). Participants are then contacted up to 4 weeks after their visit when their lab test results are available to complete the screening process.

**Table 2:** Schedule of safety and clinical assessments

Assessment name	Pre-SCR	SCR	BSL	8 w	12 w	3M	16 w	20 w	6M	9M	12M	15M	18M	As clinically prescribed
Point-of-care HIV, HCV, and pregnancy rapid testing		**X**												**X**
Hepatitis A virus test, anti-HBs, anti-HBc		**X**												**X**
Pregnancy and birth control assessment		**X**	**X**			**X**			**X**	**X**	**X**	**X**	**X**	
Medical and psychiatric history		**X**												
Physical exams and vital signs		**X**												**X**
HCV Ab, HCV RNA		**X**	**X**			**X**			**X**	**X**	**X**	**X**	**X**	**X**
Liver and kidney function		**X**	**X**											**X**
HIV, syphilis, chlamydia, gonorrhea, and hepatitis B			**X**			**X**			**X**	**X**	**X**	**X**	**X**	
Concomitant medications			**X**			**X**			**X**	**X**	**X**	**X**	**X**	
Adverse events and serious adverse events			**X**			**X**			**X**	**X**	**X**	**X**	**X**	
Treatment														
Clinical management			**X**											**X**
Dried blood spot			**X**	**X**	**X**		**X**	**X**	**X**	**X**	**X**	**X**	**X**	
HCV treated participants														
HCV RNA	**12w post-Tx initiation, 12w post-end of treatment**													

*SCR* screening, *BSL* baseline, *Tx* treatment, *w* week, *M* month

**Table 3:** Schedule of study procedures and assessments

Assessment name	Pre-SCR	SCR	BSL	3M	6M	9M	12M	15M	18M
Verbal consent and pre-screening questionnaire	**X**								
Screening informed consent form		**X**							
Locator form		**X**	**X**	**X**	**X**	**X**	**X**	**X**	**X**
Medical release authorization forms		**X**							
Injection drug use and eligibility questionnaire		**X**							
HIV and HCV testing history and readiness, knowledge		**X**							
Demographic questionnaire, health literacy		**X**							
Trial informed consent form			**X**						
Full sociodemographic survey			**X**	**X**	**X**	**X**	**X**	**X**	**X**
Substance use profile			**X**	**X**	**X**	**X**	**X**	**X**	**X**
Alcohol use, drug use			**X**		**X**		**X**		**X**
Injection profile, needle access, sharing needles and works			**X**	**X**	**X**	**X**	**X**	**X**	**X**
Overdose history survey			**X**		**X**		**X**		**X**
Attitudes towards HIV, attitudes towards HCV			**X**						
Experience of illness scale			**X**	**X**	**X**				
Medical mistrust			**X**						
History of abuse and interpersonal violence			**X**		**X**		**X**		**X**
Social support and conflictual social interaction			**X**						
Brief Pain Inventory			**X**	**X**	**X**	**X**	**X**	**X**	**X**
Brief Symptom Inventory			**X**	**X**	**X**	**X**	**X**	**X**	**X**
Household Food Survey			**X**	**X**	**X**	**X**	**X**	**X**	**X**
EuroQol-5D-5L			**X**						
Crime and legal activities			**X**	**X**	**X**	**X**	**X**	**X**	**X**
Non medical and other services			**X**	**X**	**X**	**X**	**X**	**X**	**X**
Access to care			**X**		**X**		**X**		**X**
Sexual behavior			**X**	**X**	**X**	**X**	**X**	**X**	**X**
Condom use			**X**		**X**		**X**		**X**
Communicating w/ physician about PrEP/HCV Tx self-efficacy			**X**	**X**	**X**				
Relationship with physician			**X**	**X**	**X**				
PrEP adherence HCV-PA1 and 2				**X**	**X**	**X**	**X**	**X**	**X**
Adherence counseling/patient navigation satisfaction survey	**EOI**								

*SCR* screening, *BSL* baseline, *EOI* end of intervention, *M* month

During the second screening visit, a clinician completes a physical exam and informs the potential participants of their lab results. Ineligible participants receive their lab results, proceed as they normally would for routine services, and/or receive health care and treatment based on physician recommendation.

Before study consent and randomization, eligible participants are informed of the pros and cons of being on PrEP to ensure equivalent information for all. Interest in initiating PrEP is not required to participate. Eligible participants who are still interested in participating complete the study informed consent process and sign the IRB-approved consent form.

#### Randomization

##### Randomization arm 1: on-site integrated care with adherence counseling

Participants randomized to the on-site integrated care with adherence counseling arm are offered PrEP and, if indicated, HCV treatment at the site, either the OAT clinic or SSP, where they were recruited or are receiving care/services. Nurses, physicians, and counselors trained in PrEP and HCV treatment meet with participants and provide care directly at OAT and SSP venues. For participants who choose to, the goal will be to initiate PrEP and/or, if applicable, HCV treatment as soon as possible.

Counseling for PrEP initiation and adherence and for HCV treatment is provided by the clinical counseling staff of the on-site integrated care arm. Counseling assesses PrEP and HCV treatment understanding and beliefs; medication adherence, benefits, and adherence strategies; and risk behaviors and risk reduction strategies. Counselors meet with participants to support treatment adherence, discuss barriers, and develop a personalized adherence plan.

As part of the integrated care team, the counselor conducts up to 5 face-to-face sessions over 6 months. These counseling sessions are estimated to be 20 to 35 min in duration and are tailored to each participant’s needs and risk profile. The counselor incorporates Motivational Interviewing (MI) techniques to communicate and build an effective, working relationship with the participant. MI is a patient-centered approach to counseling which recognizes that the participant is ultimately responsible for his/her own behavior change [46–51].

##### Randomization arm 2: off-site referral to specialized care with patient navigation

Participants randomized to the off-site referral to specialized care and patient navigation arm work with a patient navigator to be linked to primary care for PrEP and, if necessary, HCV treatment. We have adapted the Antiretroviral Treatment Access to Services (ARTAS) intervention, an effective linkage to care intervention for persons living with HIV, to facilitate linkage to PrEP and, if necessary, HCV treatment services for PWID [52, 53]. Participants in the off-site care arm are offered continuous PrEP and, if necessary, HCV treatment by their off-site physician.

Patient navigators actively coordinate and link participants to available clinics and community resources by scheduling appointments, arranging transportation, and assisting the participant with completing any clinic registration or health insurance enrolling/re-enrolling paperwork that a clinic or service agent may require. The intervention includes up to 5, 30–45 min face-to-face meetings with the patient navigator and participant. These meetings are tailored to each participant’s needs. Additionally, the patient navigator assists the participant in identifying and utilizing informal and formal sources of support to move along the PrEP and/or HCV care continuum, including accessing and utilizing ongoing substance use treatment and harm reduction services as needed. The patient navigator helps the participant inform off-site physicians of the trial and of the availability of PrEP and HCV medication, should the physician and patient decide to initiate one or both treatments. Brief telephone, email, and text message communication is expected between the patient navigator and care agencies/support services and between the patient navigator and the participant. These non-face-to-face contacts will be logged and tracked but will not be counted as any of the 5 face-to-face intervention meetings.

#### Provision of other medical care (both arms)

In addition to PrEP and, if indicated, HCV care, participants in the on-site integrated care arm receive a referral to ancillary care services consistent with the local standard of care. If required, the off-site physician may provide all necessary care to off-site participants.

#### Medication

Tenofovir disoproxil fumarate/emtricitabine (TDF/FTC, Truvada*®*, Gilead Sciences) and sofosbuvir/velpatasvir (SOF/VEL, Epclusa®, Gilead Sciences) are made available free of charge to participants in both arms. Clinicians are allowed to use other treatment regimens if deemed preferable for the participant.

TDF/FTC is an oral combination to be taken continuously on a once-daily basis [54]. As recommended by CDC and Canadian PrEP guidelines, participants are counseled regarding side effects and monitored every 3 months for adverse events, including signs of renal compromise.

SOF/VEL is an oral combination of sofosbuvir (NS5B inhibitor) and velpatasvir (NS5A inhibitor) [55] taken once daily for 12 weeks. SOF/VEL demonstrated high rates of sustained viral response (SVR; equivalent of cure) in phase III and real-life observational studies for all HCV genotypes [56–60].

### Criteria for discontinuing or modifying allocated interventions (11b)

#### HIV seroconversion

HIV status is monitored every 3 months. Participants who seroconvert during treatment will be immediately discontinued from PrEP and actively referred for proper HIV care.

#### Pregnancy

Participants who become pregnant will be kept in the study, but those taking TDF/FTC will be evaluated for risk by their physician to determine if TDF/FTC should be discontinued. Participants taking SOF/VEL who become pregnant during study participation will be discontinued from this medication.

#### Renal function impairment

Serum creatinine is monitored every 3 months. If a participant’s creatinine clearance decreases to <30 ml/min, serum phosphate decreases to < 1.0 mg/dl, or renal function declines with no other identifiable cause, they will be evaluated by a physician to determine if medication should be discontinued.

#### HCV treatment

HCV treatment will be discontinued in the following situation: a 10-fold increase in ALT level; an increase in ALT <10-fold that is accompanied by any weakness, nausea, vomiting, jaundice, or significantly increased bilirubin, alkaline phosphatase, or international normalized ratio (INR); and a decrease in creatinine clearance below 30 mL/min. Participants who developed decompensated cirrhosis during the trial will be immediately referred to a hepatologist who will evaluate whether treatment should be discontinued.

### Strategies to improve adherence to interventions (11c)

As part of the intervention, participants have up to 5 meetings with the adherence counselor (on-site group) or the patient navigator (off-site group). These meetings are tailored to each participant’s needs but should help them to adhere to the interventions proposed.

Adherence monitoring is based on self-report. In addition, for PrEP, liquid chromatography and mass spectroscopy will be used to detect the presence of tenofovir-diphosphate on dried-blood spots (DBS) [61, 62].

### Relevant concomitant care permitted or prohibited during the trial (11d)

Patients on HCV treatment at screening will have to postpone enrollment until after the end of treatment. Patients currently on PrEP are excluded.

Prior to enrolling in this study, participants may have pre-existing relationships with professionals for the purposes of securing housing, benefits, food, HCV care, HIV prevention, substance use, and mental health treatment. Renewed or continued contact with any such professional or paraprofessional staff may include discussion of HCV prevention and treatment, HIV prevention, and substance use treatment. Regardless of the study group, impeding any such contacts would be both unethical and infeasible. To account for non-study-related professional or paraprofessional contacts, medical records are obtained upon participant consent, and participants are asked about such exposures during follow-up assessments.

### Provisions for post-trial care (30)

Prior to study completion, research staff will connect participants with physicians and care teams in the community as appropriate or within the programs at the site where the study is conducted. Medication will be continued after the study at the patient’s discretion. Prior to study completion, research staff will attempt to connect participants with physicians and care teams in the community as appropriate or within the programs at the site where the study is conducted.

### Outcomes (12)

#### Primary outcome measures

The co-primary trial outcomes are (1) sustained PrEP adherence during the first 6 months and (2) HCV cure among participants living with HCV.

**Table 4:** Definition and measure of secondary and tertiary outcomes

Secondary outcomes	Definition	Measure
PrEP initiation/uptake	Receiving a prescription and taking the first dose of the medication	First dose of Rx dispensed to the patient and recorded at pharmacy/dispensing venue + self-report
Long-term sustained PrEP adherence	PrEP use at 6, 12, and 18 months post-randomization	Self-report + detectable level of TDF in DBS 6, 12, and 18 months post-randomization
HCV incidence/re-infection	New HCV infection	At scheduled visit: HCV-Ab positive (never infected) or detectable HCV-RNA (previously infected and cured)
STI or HIV incidence/STI re-infection	New STI or HIV infection in a previously uninfected person/previously infected and cured person	At scheduled visit: Any positive test result for *Neisseria gonorrheae*, *Chlamydia trachomatis*, syphilis, or HIV
Behavioral disinhibition	Changes or increase in sexual or injection risk behaviors	Self-report at each visit
**Tertiary outcomes**	**Definition**	**Measure**
Ongoing PrEP use	Any PrEP use during any period	Any detectable level of TDF in DBS/Self-report of use
HCV SVR within those initiating	12-week sustained viral response post-end of treatment	First dose of medication dispensed to the patient and recorded at pharmacy/dispensing venue + self-report/HCV-RNA negative at 12 weeks post end of treatment

Sustained PrEP adherence will be compared between both treatment arms at 6 months post-randomization. Sustained PrEP adherence will be defined as the self-report of achieving daily PrEP use, which will be confirmed by evidence of detectable levels of tenofovir. In order to achieve protection against HIV infection, TDF/FTC should be taken once daily to achieve protective levels. PrEP use will be self-reported by participants. Blood tenofovir will be detected using DBS collected every month beginning at 8 weeks post-PrEP initiation during the first 6 months post-randomization and subsequently every 3 months during research visits.

HCV cure is defined as initiating HCV treatment within 6 months of being randomized and achieving SVR by 12 months post-randomization. HCV treatment initiation will be measured using self-report and by assessing medical/drug dispensation records. SVR12 is defined as a negative HCV-RNA 12 weeks after end of treatment.

#### Secondary and tertiary outcomes measures

Secondary and tertiary outcomes measures are presented in Table 4.

### Participant timeline (13)

The participant timeline is presented in Fig. 1.

### Sample size (14)

We will utilize an alpha of .025 to account for our two co-primary outcomes. Our sample will include 500 participants in the PrEP component and approximately 240 in the HCV component. We estimated power using a simulation program written for SAS 9.4. This program generated data with various constellations of treatment effects, site effects, venue effects, and interactions. A simple power analysis shows that with 500 participants and alpha = .025 that there is 90% power to uncover an absolute difference of approximately 15% (40% versus 55.5%, RR = 1.40) for main effects of the treatment arm (across site and venue). This power statement assumes that response rates are at the point of highest variance in the binomial distribution—i.e., variance decreases causing power to increase if the probabilities are actually further from .5 in either direction. We anticipate larger absolute differences but have kept the simple power high to account for the potentially complex interactions we might see. For example, with this sample size, the power for the primary (PrEP) outcome is about 86.8% when there are differences in site and venue of 13% and 16% respectively, and when power for the site and venue differences is greater than 80%. There is better than 80% power for an interaction effect (e.g., site by treatment) with a rate ratio [RR(site 1)/RR(site2)] of at least 1.37. Power for effects within a site or venue is around 90% for an absolute risk difference of .22 (40% versus 62%). Note that planned tests of differences (i.e., interactions) by the biological variable, sex, would need to have a rate ratio of 1.5 to have 80% power. For HCV outcomes, the sample size is expected to be smaller (*n* = 240); for the HCV primary hypothesis, there is 90% power to uncover an absolute difference of 21% (40% versus 63%, RR = 1.58), well within the range of what we expect for this intervention [63]. This is based on the extremely low rates of PrEP currently in our candidate venues and the relatively high rate of continuation (58%) in the open-label extension of the Bangkok Tenofovir Study where PrEP was provided without cost as is the case in the current protocol [63].

### Recruitment (15)

To achieve a sample size of 500, approximately 720 potentially eligible individuals from the Miami and the Montreal sites will be invited to participate. Initially, each site was supposed to screen approximately 120 individuals every 6 months. The COVID-19 pandemics significantly affected recruitment (see specific section) and recruitment is now expected to be completed in Fall 2022.

Participants will primarily be recruited through the participating SSP and OAT clinics. Study sites have been consulted to establish the best method of informing their patients about the project and inviting potential participants to meet with study staff to determine their eligibility. Individuals interested in participating who are not patients at study SSP or OAT sites must first become patients of those sites to participate. Individuals who are not interested in hearing more about the study are noted in a recruitment ledger so the number of approaches made during the selected recruitment time can be calculated.

## Assignment of interventions: allocation

### Sequence generation (16a)

Participants are randomized to one of two treatment groups in a 1:1 ratio. Randomization at the individual level uses a permuted-block randomization scheme to ensure relative balance across time; the block size is randomly permuted to prevent study staff from guessing the block size and inferring the condition to which the next participant will be assigned. Randomization tables have been generated by study statisticians separately for venues within site and by HCV antibody status.

### Concealment mechanism (16b)

Randomization is handled centrally and administered via the Research Electronic Data Capture (REDCap) system to conceal the randomization process and group assignments until they are assigned.

### Implementation (16c)

The statistician generates the allocation sequence and this is encoded into the REDCap system. The study coordinator enrolls individuals into the study for assessment of inclusion-exclusion criteria. When the inclusion-exclusion variables have been determined and entered into the REDCap system, the condition assignment is generated.

## Assignment of interventions: blinding

### Who will be blinded (17a)

This trial is an open-label, un-blinded study. Given the pragmatic nature of the trial, participants, providers, and research staff are not blinded. Research and clinical staff are blinded to the randomization procedure to prevent predictions about participant assignment. Research and clinical staff are initially blinded to arm assignment to avoid bias, but after baseline, the research and clinical staff are not blinded.

Regarding data analysis, analysis strategies are pre-specified, and complete statistical analysis plans will be finalized prior to the data being locked. Whereas the data analysts will not be blinded because the same staff is performing data management, the study investigators including the study statistician (DJF) will be blinded to treatment assignment until results are finalized.

### Procedure for unblinding if needed (17b)

Not applicable, see 17a

## Data collection and management

### Plans for assessment and collection of outcomes (18a)

Data collection is planned at screening, baseline, and every 3 months thereafter. “Full” research follow-up visits occur at approximately 6, 12, and 18 months post-randomization while participants have short interim research visits approximately 3, 9, and 15 months post-randomization. In the first 6 months post-randomization, participants are scheduled for DBS monthly after 8 weeks of initiating treatment if on PrEP. Pregnancy tests will be required at each research visit for all randomized participants of child-bearing potential. Tables 2 and 3 describe the battery of assessments and lab assessments planned.

Assessments are administered in a private space by a trained interviewer through a computer-assisted personal interview (CAPI) or through paper case report forms (CRF) that are later entered into an electronic data capture (EDC) system. Self-report questionnaires are programmed in REDCap and administered from a password-protected website. Wherever possible, we use measures from NIDA’s Data Harmonization projects (e.g., Seek, Test, Treat and Retain [64]) or established PrEP studies (e.g., the PrEP Demo Project) [65]. Assessments are interviewer-administered; the initial assessment will take approximately 90 min and others 45–50 min.

#### Assessment of outcomes

For participants initiating PrEP, adherence verification by self-report and DBS collection begins 8 weeks post-PrEP initiation and is performed once a month thereafter for the first 6 months post-randomization and then at 3-month intervals until the end of the study.

For HCV-treated patients, HCV RNA is tested 12 weeks after treatment initiation (end of treatment), at SVR (12 weeks post-treatment completion/discontinuation), and at 3-month intervals thereafter to detect reinfection.

HIV, syphilis, chlamydia, gonorrhea, and HBV (if non-immune) testing are performed on all participants at 3-month intervals. New HCV infections are detected at 3-month intervals: HCV RNA testing is done on participants who were HCV Ab reactive and HCV RNA negative at their previous visit and HCV Ab testing is done on participants who tested HCV Ab negative at their previous visit (Table 2).

### Plans to promote participant retention and complete follow-up (18b)

The following information is collected via a locator form to minimize loss to follow-up: name, personal health number or patient identification number (depending on whether the participant has access to provincial care in Quebec or Medicaid in Florida), date of birth, the best numbers, ways, day and time to reach them, if text messaging is acceptable, email address, names of relatives and friends who would know how to reach them, and permission to contact them for that purpose, the name of the doctor or community health center that would know how to reach them, parole officer contact information (if relevant), permanent physical address (if available) alternative living situation and address, usual hangouts, access to other organizations where a message can be left. In addition, participants are compensated for their time and effort for baseline, DBS, intervention, and follow-up visits. Compensation is issued in cash, gift card, or electronic payment. Maximum compensation for participants over their 18-month participation is US $440 in the Miami sites and Canadian $455 in the Montreal sites.

### Data management (19)

Data management is coordinated using REDCap tools hosted at the University of Miami using a confidential and secure database management system that includes consistency and range checks [66, 67]. The data management team is responsible for the development and validation of the clinical study database, ensuring data integrity, and training site staff on applicable local data management procedures. To address the issue of data entry quality, the data management team follows a standard data monitoring plan. If the data management team finds incomplete or inaccurate data, a data clarification request is generated and distributed to sites for a response. An acceptable quality level prior to study data lock or closeout will be established as a part of the data management plan.

### Confidentiality (27)

All assessments, study forms, and other records are coded using research identity (RID) numbers only. All research and clinical records are stored in a secured location with limited access. Records that have personal identifiers are stored separately from research records that contain only the participants’ RID number. This includes consent forms, clinical records, and files that link participants’ names with RID numbers. The study automatically received a Federal Certificate of Confidentiality (CoC) from the National Institutes of Health (NIH) to protect against any demands for information that would identify participants. Exceptions to confidentiality for participants are disclosures required by law, including reportable disease. Participants are informed of these exceptions in the informed consent process.

### Plans for collection, laboratory evaluation, and storage of biological specimens for genetic or molecular analysis in this trial/future use (33)

Most specimen collected during this study is a regular blood test, such as CBC, that will be collected and destroyed as per local laboratory procedures. The two exceptions are the DBS for TDF/FTC detectability and a serum sample.

DBS are collected to assess the detectability of TDF/FTC monthly for the first 6 months post-randomization (commencing at 8 weeks post-PrEP initiation) and every 3 months thereafter. After collection, specimens are left to dry for 4–24 h. Dried samples are then placed in individual plastic bags with two desiccant bags and a moisture indicator cardboard. Samples are transported at 4°C and kept frozen at −70°C. Liquid chromatography and mass spectroscopy will be used to detect the presence of tenofovir-diphosphate on DBS [61, 62].

For patients who are HCV RNA positive at baseline, a serum is kept frozen at −80°C for the duration of the study. This specimen will be used to distinguish, through sequencing, between treatment failure and reinfection in case of re-occurrence of HCV RNA positivity after treatment. All specimen will be destroyed as per local procedure at the end of the study.

## Statistical methods

### Statistical methods for primary and secondary outcomes (20a)

The *primary hypothesis* is that on-site integrated care is superior to off-site referral with patient navigation in achieving higher rates of sustained PrEP adherence and/or HCV cure, measured as the proportion of participants achieving detectable levels of tenofovir in DBS in the first 6 months of follow-up and achieving HCV sustained virologic response 12 weeks post-treatment completion after initiating treatment within the 6 months post-randomization. A sub-hypothesis is that the difference in rates of sustained PrEP adherence and HCV cure with on-site integrated care relative to referral with patient navigation will be moderated by healthcare system and venue; differences between on-site versus referral will be greater in Miami vs. Montreal and in SSP vs. OAT settings. For the *primary hypothesis*, each of our two co-primary outcomes, sustained PrEP adherence and HCV cure, will be tested at an alpha rate of .025 using a Poisson regression analysis with a binary outcome to directly estimate the risk ratio associated with on-site integrated care (rather than the odds ratio estimated by logistic regression). Both co-primary hypothesis tests will be stratified by site and venue, testing for interactions of treatment effect by site and venue due to our expectation that context is important for implementation. In the absence of qualitative interaction effects, the main effects will be examined.

We will analyze long-run sustained PrEP adherence and HCV cure resulting from treatment initiated after the first 6 months, similarly, except using a repeated-measures Poisson regression implemented as a generalized estimating equation (GEE) including the periods between 6- and 18-month follow-ups. It is important to note that for all PrEP adherence analyses, any participants who become HIV positive will be counted as PrEP non adherent. For all other secondary outcomes, similar approaches will be used. PrEP initiation will be assessed as a simple binary outcome (initial self-reported use verified by prescription in medical records) and again modeled using repeated measures Poisson regression. We will examine sustained PrEP adherence among those self-reporting taking PrEP to disentangle PrEP non-engagement, inadequate PrEP use, and protective levels of PrEP use. Note that protective levels of PrEP use will be examined only among patients not on HCV medication due to norms for protective PrEP use when taken with HCV medication not having been established. We will examine HCV treatment initiation and rates of SVR among those treated (the primary analysis will include all HCV-positive randomized participants). We will examine potential rates of behavioral disinhibition as measured by changes in self-reported sexual risk (i.e., condom-less vaginal and/or anal sex), needle/works sharing, HCV infection/re-infection rates, and STI (including HIV) infection rates over the entire follow-up period. Additional analyses will examine the timing of initiation of both PrEP and HCV treatment, and whether non-engagement and discontinuation of PrEP are related to levels of (or changes in) risk.

### Interim analyses (21b)

There are no planned interim analyses for either efficacy or futility. There may be safety-focused interim looks performed (without formal statistical testing) at the regular DSMB meetings or at unscheduled times per the DSMB’s request.

### Methods for additional analyses (e.g., subgroup analyses) (20b)

#### Mediators and moderators of outcomes

The difference in sustained PrEP adherence and HCV cure with on-site care relative to referral with patient navigation will be moderated by healthcare system and venue. As part of sensitivity analyses planned for all outcomes, we will examine differences in effects (moderation) with respect to the biological variable, sex, race/ethnicity, and various social determinants of health (e.g., housing instability, food insecurity, employment, income). We will document differences in risk profiles by these same characteristics (sex, race/ethnicity) as well as site and venue. All subgroups will be evaluated with a test of the interaction of the subgroup indicator and the treatment assignment.

### Methods in analysis to handle protocol non-adherence and any statistical methods to handle missing data (20c)

Missing data are a ubiquitous problem in substance use research, primarily due to dropping out and refusal. Missing data can lead to biased estimates and reduction of power, affecting the generalizability of the study. We are making every effort to minimize the amount of missing data. In the primary analyses, we will assume that participants who have dropped out are treatment failures (i.e., are not taking PrEP and, if HCV positive, not taking HCV medications). In all cases, missingness patterns will be identified and analyses will be conducted to determine if there is differential non-death attrition by treatment arm, and if missingness is related to any of the covariates. In the case that covariates predict missingness, the data are called “Missing at Random” (MAR) [68]. GEE analyses are only appropriate if data are “Missing Completely at Random,” which means that no covariates predict the occurrence of missing data. Under the MAR assumption, the multiple imputations procedure can be used to fill in the data without artificially compressing the variance associated with the imputed data [69]. Therefore, if observed covariates predict the existence of missing data, multiple imputation will be used in a secondary analysis of the primary outcome to assess the effects of missingness on the reported results. If nonrandom missingness is of concern (Missing not at Random (MNAR)), this problem will be addressed by applying pattern-mixture, propensity score, or related models so that the effect of bias can be assessed in sensitivity analyses.

#### Qualitative interview component

To be eligible to participate in the qualitative interview component, individuals must be an enrolled participant of the RCT in either intervention arm, must have completed the first 6 months of RCT follow-up, and must be able and willing to provide informed consent.

Following a purposeful maximum sampling strategy to optimize diversity (in terms of ages, sex and gender, level of adherence, etc.), participants are selected from both arms to ensure that most experiences are represented. Qualitative interview invitations are made to selected participants after the completion of the 6-month post-randomization follow-up visit. Interviews are scheduled according to participant availability. The interview guide addresses the following topics: (1) participant’s experience with the intervention (experience with the services offered in the experimental or the control group); (2) participant’s experience with PrEP, e.g., perception of their own risks and of the usefulness of taking PrEP, beliefs (and misbeliefs) and concerns about PrEP, management of the medication (including side effects); (3) contextual-level discussion of topics such as living conditions, other health problems such as mental health issues and intoxication/severity of substance use problem, and personal resources (work, housing, finances, education, peer norms, social support, health services access and utilization); (4) inquiry about any change in risk behavior and contextual factors involved.

Qualitative interviews are conducted by trained and bilingual (English and French or Spanish) interviewers in each city. They are digitally recorded for transcription. At the end of each interview, a report is produced by the interviewer. It includes notes on the interview conditions, i.e., all the elements that should be taken into consideration in the interpretation of the interview content (date, duration, location of the interview, state of the participant, etc.). The report will also include preliminary observations as well as suggested new avenues or hypotheses to explore in future interviews [70].

In accordance with the principle of data saturation [70, 71], we will continue sampling until new interviews no longer allow the construction or verification of important concepts related to the subject of study. Up to 100 participants (approximately 50 per city) will be interviewed.

Analyses of qualitative data will be carried out separately from the quantitative component as the qualitative component is intended to explore complementary research questions. However, quantitative results will also be examined with qualitative findings which inform the analyses and interpretation of both the quantitative and qualitative results [72].

We will use an iterative approach. Analyses will start from the beginning of interviews. Using an inductive approach, we will first identify the smallest units of meanings related to the study topic for each interview. Consistencies and variations in participants’ accounts will then be examined through constant comparative analysis, a method where the data are constantly revisited after initial coding until it is clear that no new themes are emerging [73]. Throughout these steps, we will bring out the meanings, social organization and strategies involved in PrEP use, and their relationships within the participants’ behaviors and life contexts. As the analyses will be carried out for each site, a final step of the analysis process will consist of contrasting and comparing both analyzed data sets in order to identify similarities and variations across sites in regard to the individual and structural factors that influence treatment adherence and behaviors.

The analysis process will involve the development of a mixed coding grid, based on both research questions and emerging themes in order to identify recurrent themes and conceptual categories. The software NVivo [74] will be used to help in this task. The categories identified with their properties will also be compared with theoretical concepts coming from the literature on medication adherence. This will ultimately allow us to compare our findings with the results of research conducted with other populations using PrEP and with people living with other conditions requiring prevention medication.

To ensure the validity of the analysis, we will employ a double coding procedure. Specifically, the coding of the same interviews by a second individual blind to the first coding will be performed on 25% of randomly selected interviews to evaluate the concordance of the analyses [75]. The researcher responsible for the qualitative component will supervise the entire analysis process, thus allowing an analysis from three points of view, which will enhance the validity of the conclusions. Results will be discussed in meetings with the research team.

Finally, integration of quantitative and qualitative results will be achieved using the joint displays technique and team discussions. Joint displays will provide visual means that will be used to stimulate discussions about the results and interpretation [76].

#### Cost and cost-effectiveness components

An assessment of healthcare services utilization and costs occurs in parallel to research procedures involving participants. Utilization of healthcare services delivered on site is documented in study records; healthcare services utilization outside of the intervention is collected directly from participants. Resources required to deliver the interventions in each arm will be estimated using established micro-costing methods [77]. Interviews will be conducted with site staff regarding time allocation and other resources utilized for delivering the interventions; site financial staff are asked to provide information regarding shared resources (“overhead”) with the exclusion of research-related costs.

Labor unit costs are estimated based on published labor rates in each location [78, 79]. Medication costs are estimated using Federal Supply Schedule costs for the US and published costs from Canada [80, 81]. Unit costs related to healthcare utilization outside of the trial and non-labor resources required to deliver the interventions will be estimated using local available data from Miami and Montreal (when available) and/or a systematic literature review. Using the guidelines from the Second Panel on Cost-Effectiveness in Health and Medicine, an annual discounting rate of 3% will be applied to costs and results will be reported in 2021 US dollars and Canadian dollars [82].

Results of the micro-costing analyses will be reported by arm as cost per participant enrolled on an intent-to-treat basis, as well as as-treated costs per participant to examine differences between each arm and across venues and sites. We will use a multivariable generalized linear model to predict mean costs for each healthcare service category by arm, controlling for baseline participant characteristics, including baseline service utilization. We will use the mean value of bootstrapped predictions to estimate the standard error of these healthcare costs, and a two-tailed *t*-test to estimate whether there are significant differences by study arm [83]. To capture both the long-term costs and benefits of the interventions for cost-effectiveness analysis, an extended time horizon — typically 10–25 years — is required. These include the individual benefits of HCV treatment and the public health benefits of reduced onward transmission of HIV and HCV due to PrEP and HCV treatment, respectively. Results will be generated from both the healthcare and societal perspectives. A dynamic computer simulation model (allowing HIV and HCV disease transmission between infected and susceptible segments of the population) will be used that will also capture the benefits derived from the prevention of 2nd- and 3rd-order HIV and HCV transmission [84]. We build on prior experience in both HIV [85, 86] and HCV [87–89] cost-effectiveness modeling, both alongside trials [90, 91] and in population-based settings [33, 86] to extend a previously validated dynamic compartmental HIV transmission model [85, 86, 91] to also capture the individual and public health benefits of HCV treatment. This model currently captures heterogeneity according to patient demographics, HIV risk group, and HIV disease progression [85, 92]. A subroutine capturing the progression of HCV among the infected and susceptible, informed by HCV model design considerations discussed in a recent review [93], and similar to our prior applications [87–89], will be implemented to capture the natural history of HCV. HCV transmission will also be integrated with HIV transmission in estimating the forces of infection of HIV and HCV associated with the integrated care intervention versus the standard of care.

Following past research on public health responses to HIV microepidemics in 6 US cities (including Miami), the dynamic compartmental HIV/HCV transmission model will be adapted, calibrated, and validated to match the epidemiological conditions of both Montreal and Miami [94–96]. A formal evidence synthesis will be conducted for each city, incorporating local surveillance reporting and other behavioral data if available [94]. Calibration and validation targets will then be chosen, and the model will be calibrated to match the epidemiological context of each city [95].

It is important to note that the interventions being assessed — PrEP to prevent HIV acquisition and DAA treatment to cure HCV — have both individual- and population-level impacts; the latter being the reduction in the onward transmission of HIV and HCV. As such, the population impact of these interventions will be dependent on local context — specifically, the sizes of the undiagnosed HIV and HCV populations, and the underlying availability of HIV, HCV, and harm reduction services in each city [96]. The incremental cost-effectiveness of the study intervention will therefore be assessed by city, with supporting sensitivity analyses to identify the key drivers of the differences in the cost-effectiveness of the intervention by city.

All cost-effectiveness analyses will be informed by recommendations of the Second Panel on Cost-effectiveness in Health and Medicine [82] and further adhere to best practices guidelines on model design [97], and reporting [98], and the calibration and validation [99] of dynamic infectious disease models [100]. Results will be reported as incremental cost-effectiveness ratios (ICER) comparing the two interventions within each city in US$/quality-adjusted life-years (QALY) and Cdn$/QALY at prevailing exchange rates. Both one-way and probabilistic sensitivity analyses [101] will be performed, focusing on demographic, venue, and site-level differences in the costs and effectiveness of the interventions. The healthcare resource analysis will consider different scenarios for achieving sustainability of the intervention depending on organizational and environmental considerations such as site volume, capacity, availability of services, and reimbursement rates for medications and services. We will follow established guidelines for budget impact analyses [102].

### COVID-19 impact

COVID-19 impacts on M^2^HepPrEP implementation initially included a 9-month suspension in Montreal of all in-person research activities including recruitment, screening, rapid testing, specimen collection, medical exam, enrollment, interventions, and follow-up assessments by the main Montreal IRB. Miami sites were subjected to a temporary halt to research activities followed by a rapid pivot to performing research activities remotely by phone and/or video conference when possible.

Due to a national shortage of sexually transmitted diseases (STI) testing supplies across multiple manufacturers (e.g., swabs for STI tests) in the USA, the study was also temporarily unable to perform some biological assessments. Additionally, research staff in Montreal and Miami underwent required training and an extensive clearance process via their academic institutions to obtain permission to resume in-person research activities.

Implementation of M^2^HepPrEP was further challenged by the impacts of COVID-19 measures on follow-up assessments with enrolled participants. With less than 25% of enrolled participants reachable by phone or having an Internet access, executing a communication campaign to inform enrolled participants of the transition to remote data collection was necessary.

Barriers to M^2^HepPrEP implementation also included challenges specific to recruitment venue. The SSP made numerous changes and implemented various operational flexibilities in response to COVID-19. These include modifying service and logistical operations to protect both staff and clients (e.g., use of personal protective equipment, implementing temperature checks, rearranging space to accommodate social distancing recommendations, increased distribution of syringes in the field vs. at the fixed site and increased provision of services through telehealth) and temporarily reducing services (e.g., an approximate 2-month pause on HIV and HCV testing in Miami). The SSP also temporarily shifted some staff effort toward distributing PPE and more naloxone to clients in the field. The SSP in Montreal had to adjust to observed increases in overdoses on site and amplified signs of distress. OAT program IRB in Montreal suspended clinical research activities for over 12 months as clinical staff were reassigned to COVID-19 isolation units.

Researchers, SSP, OAT programs, and people with lived experience are still working to adjust to these barriers as the potential of the trial to bridge gaps in access to HCV care and PrEP is amplified by the COVID-19 pandemic.

### Plans to give access to the full protocol, participant-level data, and statistical code (31c)

The principal investigators (PIs) — Drs. Julie Bruneau, Daniel Feaster, Valérie Martel-Laferrière, and Lisa Metsch — and their research teams will comply with all aspects of the NIH policy on Releasing and Sharing of Data. In accordance with the “Obligations of grantees, contractors and partners,” they will make the de-identified data collected by the proposed protocol available for analysis to other researchers. Data will be available either 4 years after the project is complete or 3 years after the primary outcome manuscript is published, whichever comes first. They will make the data and associated documentation available to other researchers under a data-sharing agreement that provides for a commitment to (1) using the data only for research purposes and not to identify any individual participant, (2) securing the data using appropriate computer technology, and (3) destroying or returning the data after analyses are completed. Prior to executing a data-sharing agreement, all researchers interested in obtaining data from this proposed protocol should contact the PIs and provide them with a proposal explaining the analysis that they are planning to conduct and specify which data are needed to accomplish this. Prior to sharing any data, the researchers should also provide the PIs with documentation of IRB approval from their respective institution to receive and analyze the study data.

### Oversight and monitoring

#### Composition of the coordinating center and trial steering committee (5d)

There are 4 principal investigators for this study. Together, they provide leadership and oversight of the entire project, including development and implementation of all policies, procedures, deadlines, and deliverables. A project manager in each city is responsible for supervising local staff and managing day-to-day operations, including recruitment and follow-up of participants, liaising with sites, coordinating communications, and training. PIs and project managers constitute the steering committee, which oversees activities of all study components to ensure that trial quality control, data collection, and intervention implementation goals are met within the specific timelines. They discuss study progress, data analysis, IRB issues, and all administrative responsibilities on regular calls and develop plans to address and solve any problems that may arise.

#### Composition of the data monitoring committee, its role, and reporting structure (21a)

The 5 members of the DSMB were drawn from Canada, the USA, and Australia and have clinical and research experience with the study target population and knowledge of HIV prevention, HCV care and treatment, substance use, and/or substance use disorder treatment issues. They monitor study activities at both study sites (Miami and Montreal). The DSMB meets approximately every 12 months.

#### Adverse event reporting and harms (22)

Tenofovir disoproxil/emtricitabine and sofosbuvir/velpatasvir are both approved medication and are used in this study in accordance with indications recognized by the FDA and Health Canada. Nevertheless, at every contact with the study teams, participants are asked open questions regarding potential expected or unexpected harms and medical charts are systematically reviewed. Harms are coded using the Medical Dictionary for Regulatory Activities (MedDRA) [103]. An experienced medical monitor has been appointed for the two sites and reviews all AEs and SAEs for seriousness, severity, and causality during each visit with the participant. These events are subject to ongoing monitoring by the study PIs and medical monitor and are presented for DSMB review. In the event that an adverse event or otherwise untoward incident occurs as a direct result of or in the context of the project, we closely follow IRB directives and reporting policies. A report would be provided to the NIDA Project Officer per the sponsor’s requirements. We will also conduct analyses to determine whether there are any differential adverse outcomes across groups. Except for grade 1 AE, a summary of all AE and SAE will be reported in publications.

### Frequency and plans for auditing trial conduct (23)

Trained QA monitors conduct site visits to ensure study procedures are followed and study data are collected, documented, and reported in compliance with the protocol, good clinical practice and applicable regulations. The monitors audit at mutually agreed upon times, regulatory and study documents, participant safety documentation, case report forms, and corresponding source documents. The monitors work with the investigators to verify that all study personnel are trained and able to conduct the protocol appropriately.

### Plans for communicating important protocol amendments to relevant parties (e.g., trial participants, ethical committees) (25)

Any amendments to the protocol or consent materials must be approved by the institutional review board before they are implemented. Annual progress reports and local serious adverse event (SAE) reports are submitted to each IRB, in accordance with local guidelines and procedures. The informed consent form will be updated or revised whenever important new safety information is available, or whenever the protocol is amended in a way that may affect participants’ participation in the trial.

## Dissemination plans (31a)

M^2^HepPrEP trial results will be disseminated via, but not limited to, conference presentations, peer-reviewed journal articles, and dissemination of study results to participating sites, physicians, and communities. Primary outcome and cost-effectiveness analysis results will be prioritized for conference presentations and journal publications. Results will be disseminated regardless of their effect size or statistical significance.

The PIs oversee the development of the study’s publications and research dissemination plan. They have established a publication policy based on the relative scientific contributions of the PIs and key personnel. They do not intend to use any professional writers.

## Discussion

Despite the on-going HIV epidemic among PWID, there has been little PrEP implementation research among this population [104]. Numerous groups have cited the need for more research on PrEP with PWID [32, 104, 105] and specifically research on how to optimize different HIV preventive interventions and bring them to scale for PWID [22]. Although PrEP awareness might be increasing, uptake remains low. Studies from 2015 to 2016 reported awareness of 12.4–24% and uptake of 0.7–2.6% [106, 107]. In contrast, in a survey of 397 PWID from San Francisco performed in 2018, 56.7% were aware of PrEP, but only 3.0% had used it in the last 12 months [108]. The qualitative component of our study will explore barriers and enablers as perceived by patients and staff regarding PrEP initiation and adherence. Our study’s innovation is further supported by our ability to provide new and critical data on the eligibility, uptake, and adherence of PrEP among PWID in two very different environments. While prior studies have analyzed the uptake and cost-effectiveness of PrEP among MSM [109–111], more research is needed on the implications of expanding PrEP to PWID.

Minimal research has been conducted regarding the association between PrEP use and HCV infection and/or re-infection and consists mainly of case series. This is in contrast to multiple studies examining the association of PrEP with bacterial STI risk [112–114]. Better understanding of HCV infection and reinfection rates in this population has key implications for policy and clinical care. HCV incidence is also an excellent quantitative measure of injection risk of blood-borne viral acquisition.

This trial represents a novel opportunity to assess the incremental health benefits and costs of an integrated HIV and HCV care model for PWID. It will allow us to use cutting-edge techniques to jointly simulate the impact of preventing and curing multiple comorbid conditions affecting PWID using data collected in a single clinical trial, whereas in the past our models have relied primarily on data collected for single conditions even when we seek to forecast multiple disease outcomes.

## Trial status

The current protocol version use is 1.0. The study is currently recruiting (start date: September 26 2019; approximate date of recruitment completion: Fall 2022).

## Supplementary Information

**Additional file 1:** Spirit Checklist

**Additional file 2:.** World Health organization (WHO)/Trial Registration Data Set (v 1.3.1)1.

**Additional file 3:.** Consent to participate in a research study.

## Abbreviations

Ab: Antibodies
AE: Adverse event
ALT: Alanine aminotransferase
ARTAS: Anti-Retroviral Treatment and Access Study
CAPI: Computer-assisted personal interview
CDC: Center for Disease Control and Prevention
CoC: Certificate of Confidentiality
CRF: Case report form
DAA: Direct-acting antiviral
DBS: Dried blood spot
DSMB: Data and Safety Monitoring Board
EDC: Electronic Data Capture
ERC: Ethics Review Committee
GEE: Generalized Estimating Equation
GCP: Good Clinical Practice
HBV: Hepatitis B virus
HCV: Hepatitis C virus
HIV: Human immunodeficiency virus
ICER: Incremental cost-effectiveness ratios
INR: International normalized ratio
IRB: Institutional Review Board
M2HepPrEP: Miami-Montreal Hepatitis C and Pre-Exposure Prophylaxis
MI: Motivational Interviewing
MAR: Missing at Random
MNAR: Missing not at Random
MSM: Men having sex with men
NIDA: National Institute on Drug Abuse
NIH: National Institutes of Health
NS5A: Nonstructural protein 5A
NS5B: Nonstructural protein 5B
OAT: Opioid agonist therapy
PI: Principal investigator
PrEP: Pre-exposure prophylaxis
PWID: People who inject drugs
QA: Quality assurance
QALY: Quality-adjusted life-years
RNA: Ribonucleic acid
RCT: Randomized controlled trial
REDCap: Research Electronic Data Capture
RID: Research identification
RR: Risk ratio or relative risk
SAE: Serious adverse event
SOF/VEL: Sofosbuvir/velpatasvir
SOP: Standard operating procedure
SSP: Syringe service programs
STI: Sexually transmitted infection
SVR12: Sustained viral response 12 weeks post-treatment completion
TDF/FTC: Tenofovir disoproxil fumarate/emtricitabine
