# Correction: M^2^HepPrEP: study protocol for a multi-site multi-setting randomized controlled trial of integrated HIV prevention and HCV care for PWID

**DOI:** 10.1186/s13063-022-06724-9

**Published:** 2022-09-27

**Authors:** Valérie Martel-Laferrière, Daniel J. Feaster, Lisa R. Metsch, Bruce R. Schackman, Christine Loignon, Bohdan Nosyk, Hansel Tookes, Czarina N. Behrends, Nelson Arruda, Oluleye Adigun, Marie-Eve Goyer, Michael A. Kolber, Jean-Francois Mary, Allan E. Rodriguez, Iveth G. Yanez, Yue Pan, Rania Khemiri, Lauren Gooden, Aïssata Sako, Julie Bruneau

**Affiliations:** 1 Centre hospitalier de l’Université de Montréal, Montreal, Canada; 2 University of Miami Miller School of Medicine, Miami, USA; 3 Columbia University Mailman School of Public Health, New York City, USA; 4 Weill Cornell Medical College: Weill Cornell Medicine, New York City, USA; 5 Université de Sherbrooke, Sherbrooke, Canada; 6 Simon Fraser University, Burnaby, Canada; 7 Direction régionale de la santé publique de Montréal, Montreal, Canada; 8 Golden Glades Treatment Center, North Miami Beach, USA; 9 Faculté de médecine: Université de Montréal, Montreal, Canada; 10 CACTUS Montréal, Montreal, Canada; 11 University of Miami Department of Public Health Sciences, Miami, USA; 12 Centre de Recherche du CHUM: Centre hospitalier de l’Université de Montréal Centre de Recherche, Montreal, Canada

**Correction: Trials 23, 341 (2022)**

**https://doi.org/10.1186/s13063-022-06085-3**

Following the publication of the original article [1], we were notified of a typo in Dr Bruce Schackman's last name.

Originally published name: Bruce Shackman

Correct name: Bruce Schackman

The original article has been corrected.
