# Supplementary material for: M2HepPrEP: study protocol for a multi-site multi-setting randomized controlled trial of integrated HIV prevention and HCV care for PWID
Source: Trials. 2022 Apr 23;23:341. doi: 10.1186/s13063-022-06085-3 (PMC9034074; doi:10.1186/s13063-022-06085-3)
Supplement: Supplementary file 2 — Additional file 2:. World Health organization (WHO)/Trial Registration Data Set (v 1.3.1)1. [file 13063_2022_6085_MOESM2_ESM.docx]

| **World Health organization (WHO)/ Trial Registration Data Set (v 1.3.1)1** | |
| --- | --- |
| 1. Primary Registry and Trial Identifying Number | Clinicaltrials.gov  NCT03981445 |
| 1. Date of Registration in Primary Registry | June 10, 2019 |
| 1. Secondary Identifying Numbers | NA |
| 1. Source(s) of Monetary or Material Support | National Institute on Drug Abuse (NIDA) |
| 1. Primary Sponsor | Columbia University |
| 1. Secondary Sponsor(s) | Université de Montréal  University of Miami  Weill Medical College of Cornell University  Université de Sherbrooke  Simon Fraser University  National Institute on Drug Abuse (NIDA)  Centre hospitalier de l'Université de Montréal (CHUM) |
| 1. Contact for Public Queries | Valérie Martel-Laferrière, MD  Valérie Martel-Laferrière, MD, MSc, FRCPC  Microbiologist-infectious diseases specialist, CHUM  514-890-8000, ext. 20938  valerie.martel-laferriere.med@ssss.gouv.qc.ca |
| 1. Contact for Scientific Queries | Valérie Martel-Laferrière, MD  Valérie Martel-Laferrière, MD, MSc, FRCPC  Microbiologist-infectious diseases specialist, CHUM  514-890-8000, ext. 20938  valerie.martel-laferriere.med@ssss.gouv.qc.ca |
| 1. Public Title | M2 Hep PrEP |
| 1. Scientific Title | A Multi-site Multi-Setting Randomized Controlled Trial (RCT) of Integrated HIV Prevention and HCV Care for People Who Inject Drugs (PWID) |
| 1. Countries of Recruitment | Canada, United States |
| 1. Health Condition(s) or Problem(s) Studied | People Who Inject Drugs (PWID)  Hepatitis C (HCV) treatment  HIV preexposure prophylaxis (PrEP) |
| 1. Intervention(s) | **Behavioral: ARTAS Adapted Patient Navigation**  Patient navigation will provided by trained patient navigators to participants randomized to the off-site referral to specialized care arm. Patient Navigators will actively coordinate and link participants to available clinics and community resources by scheduling appointments, arranging transportation, and assisting the participant with completing any paperwork that a clinic or service agent may require. The intervention will include up to five, 30-45 minute face-to-face meetings between the patient navigator and participant. These meetings will be tailored around each participant's needs. Additionally, the patient navigator assists the participant in identifying and utilizing informal and formal sources of support to move along the PrEP and/or HCV care continuum. The patient navigator will help the participant inform off-site physicians of the trial and of the availability of PrEP and HCV medication, should the physician and patient decide to initiate one or both treatments.    **Behavioral: Adherence Counseling**  Counseling for PrEP initiation and adherence and, if necessary, HCV treatment will be provided by the clinical counseling staff of the on-site integrated care arm. Adherence counseling will include, but not be limited to, the indications, advantages, and disadvantages (e.g. side effects) of PrEP and HCV treatment in order to help the participant with his/her decision. The counselor will provide any necessary information to the participants and help them to address health and social needs. If required, the counselor will help the patient and physician with insurance-related issues. Adherence counselling will be carried out in a motivational style. The intervention will include five 30-45 minute face-to-face meetings with the participant and the adherence counselor over 6 months |
| 1. Key Inclusion and Exclusion Criteria | **Inclusion Criteria:**  Individuals must meet the following criteria to be eligible to participate in the RCT:   1. be 18-64 years of age 2. report injection drug use in the past 6-months 3. be HIV negative 4. provide informed consent 5. complete a medical release form 6. report living in the vicinity and being able to return for follow-up over 18-months 7. be willing to use a medically acceptable form of contraception throughout the study duration (for women of childbearing potential) 8. be able to communicate in English, French, or Spanish (site dependent) 9. be receiving services at an opioid agonist therapy clinic or a syringe access program   Individuals must meet the following criteria to be eligible to participate in the qualitative interview:   1. have completed the first 6 months of RCT follow up; 2. be able and willing to provide informed consent.   Exclusion Criteria:  Individuals will be excluded from the RCT if they:   1. have any disabling medical conditions as assessed by medical history, physical exam, vital signs, and/or laboratory assessments that in the opinion of the study physician preclude safe participation in the study or ability to provide fully informed consent. 2. have any disabling mental conditions as assessed by medical history and clinical assessment that in the opinion of the study physician precludes safe participation in the study or ability to provide fully informed consent. 3. have chronic renal failure 4. have or have history of decompensated cirrhosis 5. are HIV-positive or have symptoms of an acute HIV infection 6. are pregnant (verified via pregnancy test), are planning to become pregnant during the course of the study, or breastfeeding 7. have an allergy or contraindication to one of the study medications 8. have prior HCV treatment failure with direct-acting antiviral (DAA) regimens (Except those who were treated, cured the virus, but were re-infected with a new virus) 9. are currently on PrEP and/or HCV treatment. |
| 1. Study Type | Interventional |
| 1. Date of First Enrollment | September 2019 |
| 1. Sample Size | 500 participants that the trial plans to enroll in total |
| 1. Recruitment Status | Recruiting: participants are currently being recruited and enrolled |
| 1. Primary Outcome(s) | 1. sustained PrEP adherence at 6 months (Time Frame: 6-months post treatment initiation ) 2. HCV sustained viral response 12 weeks post-treatment completion (SVR12) among PWID recruited in OAT and SAP settings situated in Miami, Florida and Montreal, Quebec. |
| 1. Key Secondary Outcomes | - **Long-term sustained PrEP Adherence [ Time Frame: Up to 18 months ]**   Proportion of participants who self-report daily PrEP use and achieve protective levels of tenofovir as measured by DBS testing at 6-months, 12 months and 18 months post-baseline.   - **Behavioral disinhibition [ Time Frame: Up to 18 months ]**   Proportion of participants who increase sexual or injection risk behaviours as measured by self-report questionnaires administered at each research visit.   - **STI Incidence [ Time Frame: Up to 18 months ]**   Sexually Transmitted Infections (STI) incidence will be defined as a positive test result for *Neisseria gonorrhoeae, Chlamydia trachomatis*, and syphilis in participants who formerly tested negative for STIs.   - **HCV Incidence [ Time Frame: Up to 18 months ]**   HCV status will be determined by HCV-Ab testing, and if positive, HCV-RNA testing. New incidence of HCV will be defined as an HCV-Ab positive test results in participants who were HCV-Ab negative at a previous testing visit and HCV-Ab positive/HCV-RNA positive test results in participants who had previously tested HCV-Ab positive/HCV-RNA negative |
| 1. Ethics Review | Columbia University  Centre hospitalier d l’université de Montréal-CHUM |
| 1. Completion Date | Projected completion date January 2024 |
| 1. Summary Results | Pending |
| 1. IPD Sharing Statement | Plan to share IPD (no) |
