# Supplementary material for: M2HepPrEP: study protocol for a multi-site multi-setting randomized controlled trial of integrated HIV prevention and HCV care for PWID
Source: Trials. 2022 Apr 23;23:341. doi: 10.1186/s13063-022-06085-3 (PMC9034074; doi:10.1186/s13063-022-06085-3)
Supplement: Supplementary file 3 — Additional file 3:. Consent to participate in a research study. [file 13063_2022_6085_MOESM3_ESM.pdf]

TEMPLATE -- CONSENT TO PARTICIPATE IN A RESEARCH STUDY

Study title: M^{2}HepPrEP: A Multi-Site Multi-Setting RCT of Integrated HIV Prevention and HCV Care for People Who Inject Drugs

Study number: *[insert local IRB number]*
Participation duration: about 18 months
Anticipated number of research participants at this site: about 125
Sponsor/Supporter: National Institute on Drug Abuse (NIDA)

Researchers' contact information
Principal Investigator: *[insert local PI]*
Phone Number: *[insert local PI phone number]*
Co-Investigator/Study Coordinator: *[insert local Study Coordinator]*
Phone Number: [*insert local Study Coordinator phone number]*

KEY INFORMATION ABOUT THIS RESEARCH STUDY:

- The purpose of this study is to test the success of two (2) different methods for offering medications that prevent HIV (known as pre-exposure prophylaxis or PrEP) and cure HCV to people who inject drugs.
- The procedures in this study include: 1) taking part in interviews that ask questions about you, risk behaviors, HIV and Hepatitis C (HCV) testing and treatment, substance use, and other conditions; 2) providing blood and urine samples so we may assess HIV, HCV, sexually transmitted infections, liver function and pregnancy (as applicable); 3) signing release forms so we may look at your medical records; and 4) receiving adherence counseling and/or help from a patient navigator with getting you to a clinic for PrEP and HCV treatment (as applicable).
- The study involves up to 18 visits over a period of about 18 months. The detailed information in the "Part 1-" and "Part 2 – Study Procedures" sections below gives the full schedule and content of visits.
- The reasons you might *not* want to participate in this study are that giving blood may cause temporary discomfort from the needle stick, being testing for HIV and HCV may cause anxiety, and the medications that you may gain access to through this study may cause side effects. All of the known risks are listed in the "Risks, Stress, or Discomfort" section of this form.
- The reasons you might *want* to participate in this study are that you may learn more about PrEP and HCV medication and you may be able to reduce your risk of contracting HIV and cure your HCV (if applicable).
- Instead of volunteering for this study, you may seek PrEP and/or HCV treatment testing and treatment by yourself in the community. If you decide to take part in this study, it should be because you want to volunteer. You can choose to withdraw at any time during the study. If you choose not to volunteer, you will not lose any services, benefits, or rights you would normally have.
- If you are interested in learning more about this study, please read the details below.

You are being invited to take part in this research study because you completed screening procedures and have been found eligible to take part in the study. Before agreeing to take part in this project and to sign this informed consent form, please take your time to read, understand and consider carefully the following information. At the end, if you would like to take part in the study, you will be asked to sign this consent form.

PURPOSE OF STUDY:

- People who inject drugs (PWID) are at a high risk for contracting human immunodeficiency virus (HIV) and hepatitis C virus (HCV). If left untreated, HIV and HCV can lead to serious and life-threatening conditions.
- The purpose of this study is to test the success of two (2) different methods for offering medications that prevent HIV (known as pre-exposure prophylaxis or PrEP) and cure HCV to people who inject drugs. The study involves up to approximately 18 visits over a period of about 18 months.

Columbia University IRB
IRB-AAAR5478 (Y04M02)
IRB Approval Date: 04/14/2021
For use until: 07/07/2021

- Pre-exposure prophylaxis (PrEP) is a medication (Truvada®) that can be taken once daily to prevent a person from becoming infected with HIV if they are exposed to HIV through sex or injection drug use. HCV treatment (Epclusa®) can clear HCV from a person's blood in approximately 12 weeks.

About 500 people from approximately four (4) sites in Canada and the U.S.A. will take part in the study: two (2) in Montreal, Quebec (Canada) and two (2) in Miami, FL (U.S.A.). Each site will recruit about 125 participants. Recruitment for the study will occur over about 18 months. Persons who qualify to participate in the study and consent to participate will be expected to be part of the study for about 18 months in total.

Being part of this study is up to you. There is no penalty for not being part of this study. If you do not take part in the study, your medical care will not change at all because of your choice. After your eligibility to participate is confirmed and you consent to participate in the study, you will be asked to meet with the research team, several times over 18 months. The research team will do its best to schedule different research visits at the same time to lessen the burden on your time.

PART 1 - STUDY PROCEDURES:

Baseline Visit:

1. **Fill out Forms:** You will be asked to read and sign and/or fill out some forms during your first visit.
   - You will read and sign a consent form (this form) which will explain the study and get your permission to take part in it.
   - If you did not complete a locator information form as part of screening, you will sign a locator form, which will help us get in touch with you to remind you of the follow-up visits. The locator form will ask you for information about how to contact you for your follow-up visits. We will also ask for the contact information of another person(s) who may know how to find you if your contact information changes. If we cannot reach you with the information you provide on the locator form, we may use your medical records and/or public information found on the Internet to find updated contact information.
   - You will fill out HIPAA Authorization and/or medical record release forms as needed. This will let our staff access your medical records. We will look at these medical records to see your health care visits, treatment history and lab tests that relate to HCV and HIV. We will look at drug/alcohol treatment visits and referrals as needed. We will also look at your records to get current contact information.
     You will be offered copies of all forms to keep.

2. **Interview:** You will have a *face-to-face interview with a study staff member. The interview will ask questions about you, your health, HCV testing and treatment, HIV treatment, your sex life, and drug and alcohol use. A staff member will read questions to you from a computer. He/She will type your answers into the computer. The interview may take 2-3 hours to complete. You may skip any questions you don't want to answer. You can also stop the interview at any time.

PART 2 - STUDY PROCEDURES:
If you are determined to be HIV-negative and you are eligible for PrEP initiation, you will be randomly assigned to one of two study groups. This means that you will have the same chance of being in each of the two groups, like flipping a coin.

**Please note that neither you nor study staff can choose your study group. Also, your group cannot be changed once assigned.*

If you are in Group 1 (on-site integrated care with adherence counseling)

- You will be referred to the on-site medical team for PrEP and (as applicable) HCV treatment initiation. You will be expected to initiate your medication in front of a study clinician or authorized member of the research team

Columbia University IRB
IRB-AAAR5478 (Y04M02)
IRB Approval Date: 04/14/2021
For use until: 07/07/2021

(e.g.: Nurse or Research pharmacists). You will be provided with the standard practice counseling and prescription explanation of adequate ways to take your medication by the research staff before leaving with your medication.

- o You will be provided with one (1) monthly prescription bottle of TRUVADA®. Additionally, you will be provided with information about the medication and on how to take it for best results. For the first 24 weeks/ six (6) months, you will be asked to provide blood samples, and collect your prescription at 4-week intervals.
- o If you test positive for HCV, you will receive a 12-week prescription for EPCLUSA®. You can initiate Epclusa® at the same time as Truvada®. You will be provided with the standard practice counselling and prescription explanation of adequate ways to take your medications by the research staff before leaving with your medication. You will be provided with one (1) monthly prescription bottle of Epclusa®. You also will be provided with information about the medication and on how to take it for best results. Twelve weeks (approximately 3 months) AFTER you complete your HCV treatment, a test to verify that the HCV is no longer active in your system (SVR12) will be done.
- o (For women of childbearing potential who begin PrEP and/or HCV treatment), you will be asked to return to the study site on a monthly basis to take a pregnancy test. Pregnancy tests will occur at approximately 1, 2, 3 and 6 months after medication is started and at the discretion of clinical staff.

- You are expected to complete 5 sessions of adherence counseling within 6 months. Adherence counseling will include but not be limited to the indications, advantages, and disadvantages (e.g., side effects) of PrEP and/or HCV treatments. The counsellor will also develop a personalized adherence plan with you for your PrEP and/or HCV medication that will include scheduling, reminders, organizational skills, barriers, enablers, and social support. The adherence counseling sessions will be audio-recorded for quality assurance. The audio-recordings are to ensure that the counselor is doing their job properly and will not be used to evaluate you in any way. If you would not like your adherence counseling sessions to be recorded, you will have the option to opt out at the end of this consent form.

If you are in Group 2 (off-site referral to specialized care with a patient navigator)

- You will be referred to a trained patient navigator. The patient navigator will actively link you to available clinics and community resources for PrEP and/or HCV treatment. You are expected to complete up to 5 sessions with your patient navigator within 3 months. Patient-navigators will assist you with scheduling appointments, arranging transportation, and with completing any clinic registration or other paperwork that a clinic or service may require. The patient navigator also will *attend your first PrEP and/or HCV treatment visit with your provider. The patient-navigator will assist you in identifying and utilizing any informal and/or formal sources of support for PrEP and/or HCV care, including accessing and utilizing (as needed) ongoing substance use treatment and harm reduction services.

Follow-up Visits:

1. **Monthly/once a month every month for the first 6 months:** you will be asked to meet with the research team to retrieve your medication, to provide blood samples for dried blood spot (DBS) analysis for PrEP adherence analysis. Also, if required by your treatment (e.g. HCV) and/or if you are a woman of childbearing age, you will be asked to provide blood and urine samples for testing.
2. **Monthly/once a month every month for the remaining 12 months**: you will be asked to meet with the research team to retrieve your medication.
3. [1]**Every 3 months:** you will be asked to complete a *face-to-face confidential interview about your socio-economic status, injection drug use, mental health, sexual behavior, etc. and provide contact information via a locator information form for us to be able to contact you and schedule next visits. You will be asked to provide

[1] Exception: If you have documentation of a recent HIV, HCV or STI test, we may use those results to determine your HIV, HCV and/or STI status instead of performing the lab tests explained in this consent form.

blood, urine and saliva/oral swab (and anal swab, as needed) samples to conduct lab tests including HIV testing, HCV testing, STI testing, and a pregnancy test (for women of childbearing potential)

- The 6-month, 12-month, and 18-month visits will be approximately 90 minutes. You will be expected to complete a similar set of questions to the one you completed at baseline and provide blood, urine and saliva samples for rapid HIV and/or HCV tests, a pregnancy test (for women of childbearing potential), and other lab tests (e.g., STI testing) as done during screening and/or baseline.
- The 3-month, 9-month, and 15-month visits, will be approximately 45 minutes long. You will be expected to complete a briefer set of questions than the one completed at baseline and provide blood, urine and saliva samples for rapid HIV and/or HCV tests, a pregnancy test (for women of childbearing potential), and other lab tests (e.g., STI testing) as done during screening and/or baseline.

Receiving study medication

- ALL study participants can receive PrEP and when necessary HCV medication at no cost during their trial participation as long as they are clinically eligible to take them. ALL participants will need a prescription from a study physician to receive study medication.
- During the intervention phase, Truvada® will be prescribed once (1) for one month worth of treatment with 5 monthly renewals. During the follow-up phase, Truvada® will be prescribed once with 11 monthly renewals. Epclusa® will be prescribed once (1) for 4 week treatment with 2 renewals.

Loss of study medication bottles

- Lost and stolen bottles can only be replaced once throughout the duration of the study. Participants can only receive their prescribed bottle at the time at which they are scheduled. Study medication supply is limited. Study physicians will initiate the necessary standard protocols in the event that treatment interruption due to loss of a pill bottle that cannot be replaced through the trial could cause harm.

Qualitative Interview:

- Approximately six (6) months after your baseline assessment you may be part of the fifty (50) participants randomly invited to complete a qualitative *interview about their experience with PrEP. If you are contacted about the interview, and if would like to complete the interview, you will be asked to sign another consent form that will provide you with detailed information about the interview. If you do not want to be contacted for the qualitative interview in the future, you will have the option to opt out at the end of this consent form.

AUDIO RECORDING:

We would like to audio record the patient navigation and/or adherence counseling sessions depending on the group to which you have been assigned. If you are in Group 1, we would also like to record your sessions with the adherence counselor and if you are in Group 2, we would like to record your sessions with the patient navigator. A review of the recordings will show us if the staff member is running the session right. It will also help the study team understand what is in the sessions.

- Some recordings will be reviewed by trained members of the research team. This may include experts from other research facilities also involved in this research.
- Recordings will be identified by number only. Your name will not be recorded by the study staff or reviewer on any recordings.
- You may ask to stop being recorded at any time and can still be in the study. This will not change your care in any way.
- All recordings will be kept in locked file cabinets and/or password protected computers in a locked office. Only study staff will have access to them. Study records will be kept for at least six years.
- Information from the recordings may be published or shared in study reports. This will help describe the sessions

Columbia University IRB
IRB-AAAR5478 (Y04M02)
IRB Approval Date: 04/14/2021
For use until: 07/07/2021

and how they were conducted. Your name or other information that might reveal who you are will not be given in any reports or writings that may result from this study.

By checking "yes" and writing your initials below, you allow the researchers to record your sessions and use them for their research. If you check "yes" you may change your mind later and decide not to have your sessions recorded. If you choose not to have the sessions recorded you may still take part in the study.

______________Yes__________________________No______________________________Initials

RISKS, STRESS, OR DISCOMFORT:

- **Blood drawing risks:** Drawing blood may cause temporary discomfort from the needle stick and/or bruising. Rarely, infection or a small bump or swelling to the vein around the area may occur. All measures will be taken to lessen this risk by following the right steps for drawing blood.

- **HCV testing risks:** Being tested for HCV may cause anxiety no matter the test results. A reactive rapid HCV antibody test means that you have been infected with the HCV virus. A positive HCV RNA test means that you have active HCV that should be examined and treated. If either test is negative, there is still the chance that you could later become infected with the HCV virus and test positive at some time in the future, even if your body has cleared the infection once. There is always the chance that the test results could be wrong.

- **Medication side effects:** Participation in this study will expose you to the medication side effects listed below. The study clinicians will discuss this with you. The study clinicians will monitor you closely to see if you have any unexpected side effects. If possible, you will be given other medications to reduce them and make them tolerable. Many adverse effects disappear shortly after the end of the study treatment, but they can sometimes be serious, last a long period, be permanent or even cause death. Truvada® (PrEP) and Epclusa® (HCV treatment) are both approved by the Food and Drug Administration (FDA). The common and rare side effects of each medication are:

  Known side effects associated with Truvada®

  - Very common (reported by between 8% and 5% of patients)
    - Nausea
    - Dizziness
    - Fatigue
    - Diarrhea
    - Headache
    - Rash
  - Common (reported by 4% or less of patients)
    - Depression
    - Insomnia
    - Abnormal dreams
    - Sinusitis
    - Upper respiratory tract infections
    - Nasopharyngitis
    - Somnolence
    - Vomiting
    - Anemia
    - Decrease in weight
  - Rare (these risks will be thoroughly explained to you and monitored by the study physician)

Columbia University IRB
IRB-AAAR5478 (Y04M02)
IRB Approval Date: 04/14/2021
For use until: 07/07/2021

- Worsening of hepatitis B virus (HBV) infection
- Kidney problems, including kidney failure
- Severe liver issues
- Bone problems

Known side effects associated with Epclusa ®

- Common (reported in 3-4% of patients)
    - Headache
    - Fatigue
- Less Common (reported in less than 2% of patients)
    - Leukopenia
    - Palpitations
    - Vertigo
    - Abdominal distension, abdominal pain, abdominal pain upper, constipation, diarrhea, dry mouth, dyspepsia, flatulence, gastroesophageal reflux disease, nausea, stomatitis, tongue coated, toothache, vomiting
    - Asthenia, chest pain, edema peripheral, influenza like illness, pain, pyrexia
    - Lower respiratory tract infection, nasopharyngitis, sinusitis
    - Electrocardiogram QT prolonged, weight decreased
    - Decreased appetite, gout, increased appetite
    - Arthralgia, back pain, muscle spasms, musculoskeletal pain, myalgia, neck pain, osteoarthritis, pain in extremity, spinal pain, tendon pain
    - Disturbance in attention, dizziness, dyspepsia, migraine, psychomotor hyperactivity, somnolence
    - Anxiety, apathy, attention deficit/hyperactivity disorder, confusional state, depressed mood, depression, insomnia, irritability, loss of libido, mood swings, sleep disorder
    - Cough, dyspnea, epistaxis, oropharyngeal pain
    - Alopecia, eczema, pruritus generalized, rash, rash pruritic
    - Hypertension, hypertensive crisis, hypotension

Note: Gilead Sciences, Inc. is providing the Truvada® and Epclusa® to this study at no charge. As such, we will share safety-related data (i.e., data concerning medical side effects) with Gilead. However, these data will be de-identified (will not contain your name or other data that may reveal your identify) before being shared with Gilead.

- **Other risks:** There are no known psychological risks related with the interview questionnaires, procedures, or counseling in this study. It is possible that some sensitive topics such as HCV, HIV or substance use can make some participants feel uncomfortable. You have the right to refuse to answer any question that you do not wish to answer. There may also be risks of possible loss of privacy and confidentiality when taking part in a research study. There may be risks that are unknown.

BENEFITS:
There may or may not be any benefits to you from taking part in this study. You may benefit by going to a clinic and starting PrEP medication for the prevention of HIV infection and/or going to a clinic for HCV evaluation, care and treatment. You may also benefit from learning more about HCV and PrEP. Information learned from this study may help to improve HIV prevention efforts and HCV care for other people in the future.

ALTERNATIVES:
You have the choice not to take part in this study. If you choose not to be in this study, your choice will not affect your current medical care. You may seek PrEP and HCV treatment by yourself.

Columbia University IRB
IRB-AAAR5478 (Y04M02)
IRB Approval Date: 04/14/2021
For use until: 07/07/2021

CONFIDENTIALITY:
The results of your HCV, HIV, lab results and any treatment you may receive may become part of *[insert name of site]* records and your study records. Reactive HIV and/or HCV antibody tests and positive HCV RNA tests may be reported to the *[insert "state" or "province", as applicable]* and local health departments with information identifying you, as needed. You may ask study staff any questions about the reporting rules. All other information collected as part of this study will be part of your study records and will be kept confidential. The Food and Drug Administration (FDA) and Department of Health and Human Services (DHHS) *[insert Canadian authorities, as applicable]* may review your records to check if the study was done correctly. Authorized Columbia University and University of Miami employees or other agents will be bound by the same confidentiality rules and may also review your records for audit purposes.

To keep your information private, most of your study records will have a unique research ID number instead of your name on them. Samples sent to commercial or external labs for processing may contain your date of birth, but that will not be combined with your name. To help protect your privacy, the study researchers have obtained a Federal Certificate of Confidentiality from the National Institutes of Health. With this Certificate, the researchers cannot be forced, even by a court order, to share research information that may identify you in any civil, criminal, administrative, legislative, or other proceedings in any court.

The researchers will use the Certificate to protect against any demands for information that would identify you. If we learn about abuse of a child or elderly person or that you intend to harm yourself or someone else, or about certain communicable diseases, we will report that to the proper authorities. Also, if you commit or threaten to commit a crime on study program premises or against program staff, we may report that to the proper authorities. We will make every effort to keep your research records confidential. Records that identify you and the consent form that you signed may be looked at by:

- The National Institute on Drug Abuse (study sponsor), and its agents
- Office of Human Research Protections, part of the DHHS
- Columbia University Medical Center IRB and participating researchers
- University of Miami Miller School of Medicine Human Subjects Division and participating researchers
- University of Montreal and participating researchers
- Weill Cornell Medical College
- Simon Fraser University
- University of Sherbrooke
- Gilead Sciences, Inc.
- Local and/or *[insert "state" and/or "provincial"]* Institutional Review Boards (IRB)
- Quality assurance staff for this study
- Data and Safety Monitoring Board

[insert "State" or "Provincial"] and Federal government representatives or university staff sometimes look at studies to make sure they are being done in a safe and legal manner. Your study records may be reviewed, but they will not be used to put you at legal risk of harm. The reviewers will try to protect your privacy.

A description of this study will be available on http://www.ClinicalTrials.gov, as required by U.S. law. This Web site will not have information that can identify you. At most, the Web site will have a summary of the results. You can search this Web site at any time. Information will also be available to researchers on another Web site, https://datashare.nida.nih.gov/. This will be after the study is done and the data are analyzed. This Web site will not include information that can identify you. You can view this Web site at any time. Because this Web site will include de-identified study data, it is possible that the information collected from you in this study may be used for future research studies without additional consent from you. No blood, urine or other specimens collected from you during this study will be used for future studies without your explicit consent.

COST:
You will not be charged for the Truvada® and/or Epclusa® medication, counseling, interviews or other activities

Columbia University IRB
IRB-AAAR5478 (Y04M02)
IRB Approval Date: 04/14/2021
For use until: 07/07/2021

done as part of this study. However, you or your insurance company are responsible for all costs related to the clinical treatment that you may receive if linked to available clinics and community resources for PrEP and/or HCV treatment.

COMPENSATION:
All study participants will be paid for their time and effort in taking part in this study. Study participants may receive up to $365 in cash or gift vouchers. Payment will be made after completion of the following activities:

	Baseline visit	Monthly DBS samples	Patient navigator or Adherence counselling sessions	3, 9,15 month follow-up visits	6,12,18 month follow-up visits
Per visit	$40	$10	$10	$20	$45,$50,$60
Potential Number of times	1	6	5	3	3
Total	$40	$60	$50	$60	$155

You also will be offered a beverage and snacks during your research visits. If you withdraw from the study, or are withdrawn before it is completed, you will receive compensation according to the number of activities you have completed and you will be asked to complete a brief study completion questionnaire. Participants invited to take part in the qualitative interview will be given $50 at the end of the interview for a total of up to $440 in compensation for overall study participation (including screening).

COMPENSATION FOR INJURY:
You may be exposed to risk of injury from taking part in this study. If injury occurs, treatment will in most cases be available. If you have insurance, your insurance company may or may not pay for these costs. If you do not have insurance, or if your insurance company refuses to pay, you will be charged. Funds to compensate for pain, expenses, lost wages and other damages caused by injury are not routinely available.

SOURCE OF FUNDING:
Funding for this research study will be provided by the National Institute on Drug Abuse (NIDA).

RIGHT TO DECLINE OR WITHDRAW:
Taking part in this study is up to you. You may decide not to take part or you may leave the study at any time. Your decision will not result in any penalty or loss of benefits to which you are entitled. If you withdraw your consent for this study at any time, no further contact will be made. You may be withdrawn from the study by the investigator or sponsor at any time without your consent for various reasons. Reasons may include, but not be limited to, the study seeming to be harmful to you; you being unable to follow study procedures; or you being unable to keep study appointments. If you withdraw (or are withdrawn) from the study, we will still keep your study information and data until 6 years after study completion. During the course of the study, we will inform you of any new findings that may affect your decision to continue in the study.

DISCLAIMER:
Due to the evolving COVID-19 pandemic and the need to practice "social distancing," there may be disruption to some study activities. As needed, all in-person activities (e.g., interviews, completion of forms, consent processes, intervention sessions) may occur remotely by video call or telephone call. HIV/HCV provider and medication initiation visits may occur remotely by video call/telehealth and we may refer you to a local lab(s) to collect and process your specimens. Also, we may compensate you remotely by mailing payment through money order, providing a Visa card that our research staff will reload remotely, or through other electronic forms of payment as you complete study activities. If you are unable to accept any of these remote forms of payment, we will generate an "I owe you" receipt acknowledging that we will provide compensation in-person (cash or gift card) when the

Columbia University IRB
IRB-AAAR5478 (Y04M02)
IRB Approval Date: 04/14/2021
For use until: 07/07/2021

Federal government and our institution permits us to resume non-critical, in-person activities.

Please check all forms of payment below that you can accept. ***Please note that payment options 4 and 5 are not yet available. We are trying to set these up now. Therefore, you must select at least one form of payment from options 1-3 even if options 4 or 5 may work for you.*** By checking the box(es) and writing your initials below, you permit the researchers to compensate you via one or more of these methods.

☐ Money order (we will need a valid physical address)

☐ Reloadable Visa card (we will need a valid physical address)

☐ "I owe you" (We will provide an electronic receipt now documenting the compensation amount that you will receive in-person via cash or gift card in the future.)

☐ Electronic payment via Venmo, Zelle ApplePay, Cash App (We will need a valid cell phone number or email address to send payment AND you must have a debit card, credit card or linked bank account to the selected e-pay service to receive payment.)

- ☐ Venmo
- ☐ Zelle
- ☐ ApplePay
- ☐ Cash App

☐ Western Union (WU) cash pick-up (WU will require you to show a valid government-issued photo ID to pick up cash; available if WU locations are open.)

____________________Initials

CONTACT INFORMATION:
If you have any question about this study or this consent form, or if you have any problems or feel that you may have been harmed by being in this study, please call: *[insert name of local site PI]*, study doctor, at *[insert phone number of local site PI]*.

For questions about your rights as a research subject/participant, you may contact [*insert name of local IRB*] at [*insert phone number of local IRB*].

PARTICIPANT AGREEMENT:
I have read the information in this consent for (or it has been read to me). I have had my questions answered so that all parts of the study are clear to me. I freely consent to be in this research study.

By signing this consent form, I have not given up any of my legal rights.

Printed name of participant	Signature of participant	Date
Printed name of person obtaining informed consent	Signature of person obtaining informed consent	Date

Columbia University IRB
IRB-AAAR5478 (Y04M02)
IRB Approval Date: 04/14/2021
For use until: 07/07/2021

---Use the following only if applicable--

If this consent form is read to the participant because the participant is unable to read the form, an impartial witness not related with the research or investigator must be present for the consent and sign the following statement:

I confirm that the information in the consent form and any other written information was explained to, and apparently understood by the participant. The participant freely consented to participate in the research study.

______________________________	______________________________	____________
Printed name of Impartial Witness	Signature	Date

Note: This signature block cannot be used for translations into another language. A translated consent form is necessary for enrolling participants who do not speak English.

Columbia University IRB
IRB-AAAR5478 (Y04M02)
IRB Approval Date: 04/14/2021
For use until: 07/07/2021
